# Long-Term Outcomes in Patients with Locally Advanced and Metastatic Non-Small Cell Lung Cancer with High PD-L1 Expression

**DOI:** 10.3390/curroncol32040229

**Published:** 2025-04-15

**Authors:** Vesna Ćeriman Krstić, Ivan Soldatović, Milija Gajić, Natalija Samardžić, Ruža Stević, Nikola Čolić, Katarina Lukić, Biljana Šeha, Damir Radončić, Slavko Stamenić, Milan Savić, Vladimir Milenković, Brankica Milošević Maračić, Dragana Jovanović

**Affiliations:** 1 Faculty of Medicine, University of Belgrade, 11000 Belgrade, Serbia; 2 Clinic for Pulmonology, University Clinical Center of Serbia, 11000 Belgrade, Serbia; 3 Institute of Medical Statistics, Faculty of Medicine, University of Belgrade, 11000 Belgrade, Serbia; 4 Center for Radiology, University Clinical Center of Serbia, 11000 Belgrade, Serbia; 5 Clinic for Neurosurgery, University Clinical Center of Serbia, 11000 Belgrade, Serbia; 6 Clinic for Thoracic Surgery, University Clinical Center of Serbia, 11000 Belgrade, Serbia; 7 Institute of Oncology and Radiology Serbia, 11000 Belgrade, Serbia; 8 Internal Medicine Clinic “Akta Medica”, 11000 Belgrade, Serbia

**Keywords:** NSCLC, immunotherapy, pembrolizumab, long-term outcomes, PFS, OS

## Abstract

Before the introduction of targeted therapy and immunotherapy, patients with metastatic non-small-cell lung cancer (NSCLC) had a 5-year overall survival (OS) rate of up to 10%. After the positive results of KEYNOTE-024, pembrolizumab was approved in a first-line setting for patients with metastatic NSCLC and PD-L1 ≥ 50%. A small number of patients had a durable response to immunotherapy, and so far it has not been discovered who will benefit. The aim of this study was to investigate the efficacy of first-line pembrolizumab in patients with locally advanced and metastatic NSCLC with high PD-L1 expression in a real-world setting. We enrolled 35 patients with locally advanced and metastatic NSCLC who had PD-L1 ≥ 50%. Progression-free survival was 9 months, 95% CI (2.6–15.4). Overall survival was 14 months, 95% CI (0–28.5). Five-year OS rate for the whole group of patients was 20%, and the six-year OS rate was 17.2%. Immunotherapy was a revolution in the treatment of NSCLC. We still do not know which patients will benefit from immunotherapy, but patients who do respond may experience long-term outcomes.

## 1. Introduction

Prior to the development of molecular therapy and immunotherapy, patients with metastatic non-small-cell lung cancer (NSCLC) had a 5-year overall survival (OS) rate of up to 10% [1]. After the introduction of targeted therapy and immunotherapy treatment landscape has changed [1]. So far, a variety of biomarkers have been investigated in order to identify which patients could benefit the most from immunotherapy. Programmed cell death-ligand 1 (PD-L1) is the most used biomarker, and in some circumstances tumor mutation burden (TMB) is also used [1]. After the positive results of KEYNOTE-024, pembrolizumab was approved in a first-line setting for patients with metastatic NSCLC and PD-L1 ≥ 50% [2]. But it was shown that despite high PD-L1 expression, a majority of patients do not benefit from immunotherapy, or they have disease progression at some point in time [1,3]. A small number of patients have a durable response to immunotherapy, and so far it has not been discovered who will benefit [1,3]. Also, some patients with negative PD-L1 expression may benefit from immunotherapy, possibly due to intratumor heterogeneity [1,4].

Immunotherapy may not be beneficial for all patients with high PD-L1 expression, as was previously indicated. Some of them have accelerated disease progression, namely, hyperprogression (HPD) [5]. The criteria for defining hyperprogression in immunotherapy (HPD) are based on the following three parameters: tumor growth rate, tumor growth kinetics, or length of time to treatment failure [5]. The currently accepted definition made by Kato et al. is as follows: time to treatment failure of less than 2 months, tumor load increased by more than 50% compared to baseline, or cancer growth rate is more than doubled compared to the previous rate [5,6]. The incidence of HPD varies among different cancer types, and it ranges from 5 to more than 40% [5,7]. Results of different studies showed that HPD could be associated with older age, female sex, and two or more metastatic sites [5]. There is also some evidence about the association of some biomarkers with HPD [5].

This trial aimed to determine if first-line pembrolizumab was effective in treating patients with locally advanced and metastatic non-small-cell lung cancer (NSCLC) who had high PD-L1 expression, in a real-world setting.

## 2. Materials and Methods

### 2.1. Patients and Data Collection

We enrolled 35 patients treated with first-line pembrolizumab who had locally advanced or metastatic NSCLC with PD-L1 ≥ 50%. All included patients received therapy at the Clinic for Pulmonology, University Clinical Center of Serbia, and they all received pembrolizumab. One patient was treated with pembrolizumab for two years, whilst all other patients were treated until progression of the disease or death, whatever happens first. Every patient satisfied the following requirements for inclusion: histopathologically confirmed NSCLC, no EGFR and ALK mutations, PD-L1 expression ≥ 50%, locally advanced or metastatic stage of disease, had measurable disease at baseline, had never received treatment for locally advanced/metastatic disease, and had an ECOG PS of 0 or 1. Exclusion criteria were as follows: histopathologically verified other subtypes of lung carcinoma, early stage of disease, PD-L1 expression < 50%, patients who were not treatment naïve, and an ECOG PS ≥ 2. In order to determine the disease stage at baseline, a computed tomography of the chest and head or a magnetic resonance of the head were performed on all patients. The data that were collected are as follows: sex, age, smoking status [i.e., smokers, non-smokers, and ex-smokers (patients who quit smoking a year prior to treatment)], stage of the disease, response to treatment (ORR), progression-free survival (PFS), and OS. Response to treatment was defined as one of the following: complete response (CR), partial response (PR), stable disease (SD), progression of disease (PD), or hyperprogression (HPD). PFS was calculated from the initiation of pembrolizumab until disease progression or death, and OS was calculated from the initiation of pembrolizumab until death from any cause. A period of treatment failure of less than two months was considered hyperprogression. This non-interventional, observational study was conducted in accordance with the Declaration of Helsinki, and it was approved by the local Institutional Board.

### 2.2. Statistical Analysis

Depending on the type of data, results are displayed as mean ± standard deviation or count (%). The parametric t-test and the nonparametric Pearson’s chi-square test were used to compare the groups. To evaluate survival and group differences in terms of survival, the Kaplan–Meier with log-ranks test was used.

The median (95% CI) or percentage of participants who did not experience an event of interest within a certain time period is used to display survival. Every *p*-value below 0.05 has been deemed significant. We analyzed all data using SPSS 29.0 (IBM Corp. Released 2023. IBM SPSS Statistics for Windows, Version 20.0. Armonk, NY, USA: IBM Corp.).

## 3. Results

We enrolled 35 patients with locally advanced and metastatic NSCLC who had PD-L1 ≥ 50%. Main baseline demographic characteristic of all the included patients, and for patients with and without HPD, have been presented in Table 1.

In our study, 22.9% of patients had HPD. Although there was an age difference between individuals with and without HPD, it was not statistically significant. Both patients with liver metastases had HPD (*p* = 0.047). None of the patients with brain metastases had HPD, and two patients with locally advanced disease had HPD. Regarding the number of initial sites of metastases, two of them had multiple sites of metastases, another two patients had two metastatic sites, and another two of them had one metastatic site. Patients with HPD all had PD-L1 ≤ 70. They were all smokers, except for two who were former smokers. One of them received further therapy and lived for 15 month. Another seven patients died within 7 months of the pembrolizumab initiation.

At the time of the data cut-off, 17.2% of patients were still on the treatment.

**Figure 1:**
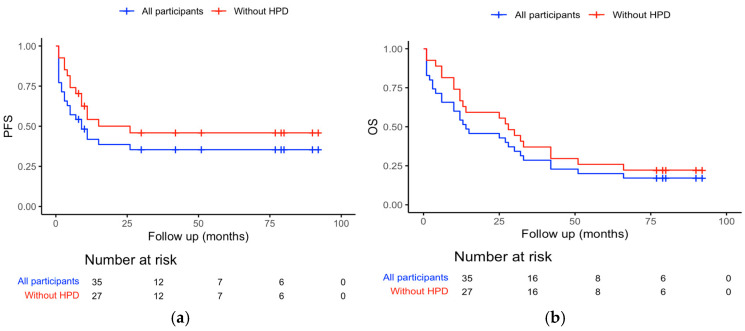
(**a**) Shows the Kaplan–Meier curve for PFS for the whole group of patients and for patients without HPD, and (**b**) shows the Kaplan–Meier curve for OS for the whole group of patients and for patients without HPD.

Progression-free survival was 9 months, 95% CI (2.6–15.4). Overall survival was 14 months, 95% CI (0–28.5). When we excluded patients with HPD, PFS was 15 months, 95% CI (0–34.3), and OS was 28 months, 95% CI (19.5–36.5). This can be seen in Figure 1a,b.

Two-year PFS and OS rates were 32.3% and 31.4%, respectively, and when patients with HPD were excluded the two-year PFS and OS rates were 42.5% and 41%, respectively. The five-year OS rate for the whole group of patients was 20%, and when patients with HPD were excluded the five-year OS rate was 25.9%. The six-year PFS and OS rates were 17.6% and 17.2%, respectively, and when patients with HPD were excluded the six-year PFS and OS rates were 23.1% and 22.3%, respectively.

Furthermore, we compared characteristics of patients who lived more than two years with those who lived less than two years. There was no significant difference between them regarding age, PD-L1 expression, gender, smoking status, histopathology, and initial stage of the disease.

## 4. Discussion

We evaluated the efficacy of first-line pembrolizumab in 35 patients with locally advanced and metastatic NSCLC with high PD-L1 expression in a real-world setting.

The KEYNOTE-024 [8] study investigated the efficacy of pembrolizumab compared to chemotherapy in previously untreated patients with metastatic NSCLC and PD-L1 ≥ 50%, but without EGFR and ALK mutations. According to the study design, patients were treated with pembrolizumab for 2 years (35 cycles) [8]. Two-thirds of patients who were initially treated with chemotherapy received immunotherapy in their further treatment course [8]. The median OS was 26.3 months and the 5-year OS rate was 31.9% [8]. The results showed that the OS for patients who had immune-related adverse events which led to treatment discontinuation was 35.9 months and the 5-year OS rate 35.1% [8]. The median PFS was 7.7 months and 3- and 5-year PFS rates were 22.8% and 12.8%, respectively [8]. Among patients treated with pembrolizumab, 39 of them (25.8%) completed 2 years of treatment [8]. This group of patients was similar to the overall study population [8]. Among them, 82.1% had a response to therapy [8]. Furthermore, after 5 years of follow-up, 32 of 39 patients were still alive [8]. The five-year OS rate for this group of patients was 81.4%, and 46.2% of patients did not have disease progression [8]. Patients with untreated brain metastases were not included in the study [8].

The KEYNOTE-042 [9] study analyzed the efficacy of pembrolizumab in previously untreated patients with metastatic NSCLC and PD-L1 ≥ 1%. In the subgroup analysis, results showed the benefit of pembrolizumab in patients with PD-L1 ≥ 50% compared to the control group [9]. The OS was 20 months for patients treated with pembrolizumab compared to 12.2 months in a control group [9]. PFS was 6.5 months [9]. The five-year OS rate was 21.9% in the pembrolizumab group compared to 9.8% in the chemotherapy group [9]. The HR for OS was 0.68 (95% CI, 0.57–0.81). In a group of patients with PD-L1 1–49%, the HR for OS was 0.88 (95% CI, 0.75–1.04) [9]. The five-year OS rate in this group of patients was 11.9%, compared to 7.4% in the group of patients treated with chemotherapy [9]. Also, we should remember that 23% of the patients treated with chemotherapy initially received immunotherapy in the subsequent treatment lines [9].

In the KEYNOTE-001 [10] study, after 5-years of follow-up the results showed that the median OS for patients treated with pembrolizumab in a first-line setting and with high PD-L1 was 35.4 months and the 5-year OS rate was 29.6%.

Rittiber et al. [11] conducted a retrospective study that included 718 patients with metastatic NSCLC and high PD-L1. The median follow-up was 16 months, and the median time on treatment was 4.4 months [11]. Twenty-one percent of patients did not progress on pembrolizumab [11]. The median OS was 14 months, but in the group of patients with an ECOG PS 0–1 it was 19.6 months and in patients with an ECOG PS ≥ 2 it was 6.1 months [11]. This study included almost 40% of patients with an ECOG PS ≥ 2, and 9% of patients had brain metastases [11].

Aguilar et al. [12] investigated the efficacy of first-line pembrolizumab in patients with metastatic NSCLC and high PD-L1 expression. The PFS was 6.5 months, and the OS was not reached [12]. But they included patients with an ECOG PS ≥ 2 (18.2%) [12].

Tamiya et al. [13] conducted a retrospective study that included 213 patients with locally advanced and metastatic NSCLC with high PD-L1 expression. One-fifth of patients had an ECOG PS ≥ 2 [13]. The PFS was 8.3 months and the OS 17.8 months [13]. However, the PFS for patients with an ECOG PS 0–1 was 9 months and for patients with an ECOG PS ≥ 2 it was 4 months [13].

Cavaille et al. [14] analyzed the efficacy of pembrolizumab in 38 patients with metastatic NSCLC and high PD-L1. The PFS was 6 months, and the OS was 11.08 months [14]. The worse results compared to the registration study could be explained by the fact that this study included 36.7% patients with brain metastases, and 26.8% patients with an ECOG PS ≥ 2 [14].

Alessi et al. [15] investigated the outcomes in patients with metastatic NSCLC and high PD-L1 expression who had ECOG PS 2 and who were treated with pembrolizumab. Among 234 patients, 16.7% had ECOG PS 2 [15]. The PFS for the whole group was 6.2 months and the OS was 19.8 months [15]. Patients with ECOG PS 2 had a significantly lower ORR (43.1 vs. 25.6%), PFS (6.6 vs. 4 months), and OS (20.3 vs. 7.4 months) compared to patients with an ECOG PS 0–1 [15]. After disease progression, this group of patients was less likely to receive second-line treatment [15].

A French retrospective study evaluated the efficacy of pembrolizumab in 108 patients with locally advanced and metastatic NSCLC with high PD-L1 expression [16]. A total of 23.1% of patients included had ECOG PS 2 and 9.2% of patients had untreated brain metastases [16]. The median PFS was 10.1 months, and the 6-month OS rate was 86.2% [16].

Descourt et al. [17] included 845 patients with advanced NSCLC and high PD-L1 expression. Among them, 20.8% had brain metastases [17]. The median PFS for the whole group was 8.2 months and the median OS was 22.6 months [17]. The twelve-month OS rate was 64.8%. The results did not differ significantly between patients with and without brain metastases, but there was a numerical advantage in the group of patients with brain metastases [17].

Cortellini et al. [18] analyzed the efficacy of pembrolizumab in more than 1000 patients with metastatic NSCLC and high PD-L1 expression. A total of 17.4% of patients had ECOG PS 2–3 and 17.6% had brain metastases [18]. A total of 15.4% of patients had liver metastases [18]. The PFS was 7.9 months, and the OS was 17.2 months [18].

Similar results were noted in other real-world studies and majority of them included patients with ECOG PS ≥ 2 and also patients with brain metastases [19,20,21].

Table 2 shows the outcomes for patients in some studies, who were treated with pembrolizumab in the first-line setting.

The study conducted by Cramer-van der Welle et al. [22] compared outcomes in patients treated with immunotherapy in real-world settings and in clinical trials. They evaluated efficacy of pembrolizumab in 84 patients in first-line settings and nivolumab in second-line settings [22]. In both settings, the PFS was comparable between real-world results and trials (8.9 months for the first-line pembrolizumab), but the OS was significantly shorter in the real-world setting for first-line pembrolizumab (15.6 months, 12-month OS rate was 57%). Also, patients in the real-world setting were less likely to received subsequent treatment [22].

We found in our study the incidence of HPD to be 22.9%. According to our knowledge, this is the first study that reported the incidence of HPD in a first-line setting. The incidence in other studies varies from 5%, to more than 40% in some studies with melanoma patients [5,6,7,23,24,25,26,27,28,29,30,31,32,33,34]. However, the definition of HPD was different among studies, and all of those studies reported the incidence of HPD in previously treated patients. Table 3 shows the incidence of HPD in patients treated with immunotherapy (PD-1/PD-L1 inhibitors, CTLA-4 inhibitors) in some of the studies which included patients with NSCLC, and some of the studies which included multiple tumor types including NSCLC.

The limit of our study is a small number of patients. The PFS in our study was comparable with the results of the real-world studies and the registration study. But the OS was shorter compared to the registration study and similar to the OS in real-world studies, possible due to a high percentage of patients with HPD. When we excluded patients with HPD, the OS doubled, and it was comparable with results in the registration study. Furthermore, when we analyzed the characteristics of patients with and without HPD, we found that patients with HPD were older compared to patients without HPD, but the difference was not significant. Interestingly, none of the patients with brain metastases had HPD, which may suggest the effects of immunotherapy in patients with brain metastases, unlike in patients with liver metastases (both of them had HPD, *p* = 0.047). Also, a difference was found in metastases in the adrenal glands (*p* = 0.030). But those results should be interpreted with caution due to the small number of patients.

## 5. Conclusions

Immunotherapy has made a revolution in the treatment of NSCLC. We still do not know which patients will benefit from immunotherapy, but patients who do respond may experience long-term outcomes.

## Figures and Tables

**Table 1:** Main baseline demographic characteristics of all included patients, and patients with and without HPD.

Demographic Characteristics	All Patients (*n* = 35)	Patients Without HPD (*n* = 27)	Patients with HPD (*n* = 8)	*p* Value
Age, mean ± sd	61.5 ± 6.9	60.96 ± 6.11	63.37 ± 9.26	0.391
Gender				0.685
Female sex, *n* (%)	11 (31.4%)	8 (29.6%)	3 (37.5%)	
Male sex, *n* (%)	24 (68.6%)	19 (70.4%)	5 (62.5%)	
Smoking, *n* (%)				0.664
Non-smokers	2 (5.7%)	2 (7.4%)	0 (0%)	
Former smokers	13 (37.1%)	11 (40.7%)	2 (25.0%)	
Current smokers	20 (57.1%)	14 (51.9%)	6 (75.0%)	
Tumor histologic type				0.117
Adeno	29 (82.9%)	24 (88.9%)	5 (62.5%)	
Squamous	6 (17.1%)	3 (11.1%)	3 (37.5%)	
Stage				0.431
IIIb/c	14 (40%)	12 (44.4%)	2(25.0%)	
IV	21 (60%)	15 (55.6%)	6 (75.0%)	
Sites of metastasis initially				
Lungs	13 (37.1%)	8 (29.6%)	5 (62.5%)	0.116
CNS	4 (11.4%)	4 (14.8%)	0 (0%)	0.553
Pleura	2 (5.7%)	1 (3.7%)	1 (12.5%)	0.410
Liver	2 (5.7%)	0 (0%)	2 (25%)	0.047
Adrenal gland	4 (11.4%)	1 (3.7%)	3 (37.5%)	0.030

**Table 2:** The overview of the outcomes for patients treated with pembrolizumab in first-line setting in some studies.

Study	PFS (Months)	OS (Months)	Patients with Brain Metastases (%)	Patients with ECOG PS ≥ 2 (%)
KEYNOTE-024 [8]	7.7	26.3	11.7%	0
KEYNOTE-042 [9]	6.5	20	5.5% for whole group	0
Rittiber et al. [11]	4.4	14	9%	App. 40%
Aguilar et al. [12]	6.5	Not reached	No data	18.2%
Tamiya et al. [13]	8.3	17.8	No data	19.7%
Cavaille et al. [14]	6.0	11.08	36.7%	26.8%
Alessi et al. [15]	6.2	19.8	27.4%	16.7%
PEMBREIZH [16]	10.1	/	9.2%	23.1%
Descourt et al. [17]	8.2	22.6	20.8%	NA
Cortellini et al. [18]	7.9	17.2	17.6%	17.4%
Tambo et al. [19]	6.1	Not reached	No data	22.1%
Ivanović et al. [20]	9.3	Not reached	15%	11%
Jiménez Galán et al. [21]	3.9	7.9	15.9%	36.3%
Ćeriman Krstić et al.	9.0	14	11.4%	0

**Table 3:** Incidence of HPD in some studies.

**Study**	**Incidence of HPD**
Champiat et al. [23]	9.2%
Kato et al. [6]	5.9%
Ferrara et al. [24]	13.8%
Kanjanapan et al. [25]	6.6%
Kim CG. et al. [26]	19%
Kim Y. et al. [27]	14.3%
Lo Russo et al. [28]	25.7%
Matos et al. [29]	10.7%
Ten Berge et al. [30]	6.9%
Tunali et al. [31]	6.6%
Arasanz et al. [32]	17.9%
Petrioli et al. [33]	6.4%
Ruiz-Patiňo et al. [34]	19.8%
Ćeriman Krstić et al.	22.9%

## Data Availability

The data presented in this study are available upon reasonable request from the corresponding author (accurately indicating status).
